# Comprehensive guide for optimizing octopus immune cell preparation to enhance single cell RNA sequencing success

**DOI:** 10.1038/s41598-026-47700-6

**Published:** 2026-04-10

**Authors:** M. M. Costa, I. Ferreirós-Vidal, A. Salas, S. Dios, F. Gambón, C. Gestal

**Affiliations:** 1 Instituto de Investigaciones Marinas (IIM), CSIC, Vigo, Spain; 2 Genética de Poblaciones en Biomedicina (GenPoB) Research Group, Facultade de Medicina, Instituto de Ciencias Forenses, Instituto de Investigación Sanitaria (IDIS), Unidade de Xenética, Universidade de Santiago de Compostela, Hospital Clínico Universitario de Santiago (SERGAS), 15706 Galicia, Spain; 3 Genetics, Vaccines and Infections Research Group (GENVIP), Instituto de Investigación Sanitaria de Santiago, Santiago de Compostela, Galicia, Spain; 4 Centro de Investigación Biomédica en Red de Enfermedades Respiratorias (CIBER-ES), Madrid, Spain; 5 Department of Immunology, Vigo University Hospital Complex, Vigo, Spain

## Abstract

The common octopus, *Octopus vulgaris*, is renowned for its advanced nervous system and complex behavior, yet its immune system remains poorly understood despite its relevance for health and pathogen resistance. Understanding octopus immunity is key to sustainable aquaculture and animal welfare. Single-cell RNA sequencing (scRNA-seq) provides high-resolution insights into immune cell diversity but requires viable single-cell suspensions, which are challenging in octopus hemocytes due to aggregation, high salinity, and chemical constraints. This study presents an optimized protocol for isolating circulating hemocytes and white body-resident hemocytes (WBH) from *O. vulgaris*. Hemocytes resuspended in Marine Antiaggregant Solution (MAS) maintained > 90% viability and structural integrity for at least 2 h post‑extraction, while WBH were dissociated using combined mechanical and enzymatic methods. Cell counting comparisons revealed poor correlation between LUNA-FL and flow cytometry, whereas Neubauer chamber counts aligned closely with cytometry data. Furthermore, a reduced-EDTA MAS medium (MAS low) proved compatible with 10 × Genomics chemistry, enabling successful GEM generation, reverse transcription, and cDNA library construction. Shallow sequencing confirmed recovery of high-quality transcriptomes and distinct hemocyte populations, demonstrating the feasibility of scRNA-seq in this non-model marine species. These findings provide the first methodological framework for applying scRNA-seq to octopus immune cells, enabling detailed analysis of hemocyte diversity and function. This pipeline establishes the foundation for investigating immune responses and adaptation and offers a valuable tool for extending single-cell applications to other marine invertebrates, enhancing immunity understanding of cephalopod immunity, and supporting animal welfare and sustainable aquaculture practices.

**Supplementary Information:**

The online version contains supplementary material available at 10.1038/s41598-026-47700-6.

## Introduction

Mollusks, the second-largest phylum of invertebrates, exhibit remarkable diversity in form, habitat, and ecological roles. Many species have economic and biomedical importance and serve as models in physiological, behavioral, and neurobiological research^1–4^. Others, such as mussels, clams, oysters, abalones, and cephalopods like squids and octopuses, are key food sources^5^. Oysters and mussels have long been farmed, while abalone and cephalopod aquaculture, e.g., *Octopus maya* and *O. sinensis*, are more recent but expanding^6,7^. In Europe, *O. vulgaris* stands out as a promising aquaculture candidate due to its short life cycle, rapid growth, adaptability, high protein content, and rising demand driven by declining wild populations^8,9^. Although its life cycle has been closed experimentally^10,11^, large-scale farming remains a challenge, requiring further research and technology. Several studies have focused on optimizing their cultivation by improving animal health, particularly through the characterization of immune responses, stress‑related biomarkers, and health‑associated molecular signatures in paralarvae and adults^12–14^. As cephalopods gain attention for sustainable aquaculture, understanding their defenses against environmental and pathogenic stressors is critical. The immune system is central to survival and adaptation under farming conditions, where high densities and variable water quality increase pathogen risks. A robust immune response is vital to prevent disease and supports adaptation. A deeper understanding of cephalopod immunity is crucial for advancing science, improving animal welfare, and promoting sustainable aquaculture practices^15^.

The immune response in mollusks, and specifically in the common octopus, is mainly mediated by hemocytes, immune cells that act alone or with humoral factors in the hemolymph to fight infection^16^. These cells use strategies such as producing reactive oxygen and nitrogen species^17^, recognizing pathogen-associated molecular patterns, activating immune-related gene pathways, and secreting effector proteins^14,16,18–21^. The circulation of hemocytes within a closed circulatory system (a novelty that is unique to cephalopods among all mollusks), combined with the presence of a hematopoietic organ (a soft organ surrounded by the optic nerve), the white body^22–24^, along with other innovative and evolutionary traits^25^, underscores the sophistication of their immune system. It is reasonable to hypothesize that octopus immune responses are more specialized than in other molluscs, emphasizing the need to study their immune architecture in detail. Up to three hemocyte types have been described by classical microscopy^16,26,27^, but it is unclear whether these are distinct cell types, developmental stages, or specialized subsets. Resolving this requires methods that capture cell-level diversity.

In this context, single-cell RNA sequencing (scRNA-seq) emerges as a powerful technique for studying gene expression at the individual cell level. Unlike bulk RNA-seq, it uncovers cell heterogeneity within samples^28^. Its advantages include revealing differences between cells of the same type, discovering new cell types and states, and expanding knowledge of cellular diversity across tissues and organisms^29,30^. However, scRNA-seq also poses challenges: high cost, complex data analysis, and the need for high-quality samples. Since its development in 2009^31^, costs have dropped significantly^32^ thanks to platforms like Drop-seq and 10 × Genomics Chromium, which use microfluidics and multiplexing to improve performance and accessibility^33–35^. Still, scRNA-seq data are far more complex than bulk RNA-seq, requiring advanced bioinformatics tools and real-time updated databases^36,37^. Many of these resources adopt open-source approaches to ensure rapid dissemination and reproducibility.

Although advances in platforms, lower costs, and accessible bioinformatics tools have mitigated major challenges, preparing a high-quality single-cell suspension remains essential for reliable results, regardless of the technology used^38^. This step is widely considered the most critical -and hardest- bottleneck, requiring strict quality control. Beyond post-sequencing checks to exclude damaged cells, doublets, or environmental RNA, the starting material must meet strict requirements for efficient library preparation: no aggregates, high viability, optimal concentration, good RNA integrity, and absence of RT inhibitors^39^. These conditions are critical for cell capture and scRNA-seq performance. Platforms provide best-practice guidelines, but most are designed for mammalian cells, where recommended buffers may not suit marine cells. High salinity, in particular, can affect reaction chemistry and alter RNA profiles.

Single-cell protocols have been applied to marine invertebrates^40^, including studies in *O. bimaculoides* and *O. vulgaris* on neural diversity^41,42^. However, scRNA-seq has not yet been used for cephalopod immune cells, nor is there a tailored workflow for marine invertebrates. Designing a robust, reproducible protocol for diverse cell types is essential. Applying it to immune cells would fill a major gap, given their role in defense, homeostasis, and adaptation. Hemocytes differ markedly from neural cells, so brain-optimized protocols may not be transferable. Developing a dedicated protocol for *O. vulgaris* hemocytes represents both a technical advance and a step toward understanding cephalopod immunity. While immune-cell scRNA-seq exists for other invertebrates^43–46^, this study provides the first optimized pipeline for *O. vulgaris*. Although full protocol transferability is unlikely, our recommendations and insights may guide work on immune cells in other marine species, contributing to invertebrate immunology beyond this application.

## Methods

### Animal sampling and care

A total of two specimens of *O. vulgaris* (measuring 2 kg on average) were collected by traps, an artisanal fishing gear used by local fishermen from the Ría of Vigo, Spain (24° 14.09′ N, 8° 47.18′ W). Animals were properly transported to the Experimental Aquarium Facilities of IIM-CSIC (center for the breeding and use of experimental animals under the REGA code ES360570202001). Individuals were maintained in 500 L tanks of filtered aerated seawater at 15 ± 1 °C with a continuous recirculating flow. The photoperiod was 12 h light:12 h dark, and cleaning and physical–chemical parameter checks were performed daily. They were fed daily with frozen fish and fresh mussels. Daily monitoring of additional welfare and health parameters was conducted in accordance with Fiorito et al.^15^, using an adapted daily care checklist (personal comm.). Before the experimental trial, octopuses were acclimated for 1 week. All animals were individually stabled due to their territorial nature. Only clinically healthy adult octopuses were included in the study. Animals showing signs of disease, stress, or abnormal behavior would have been excluded from the experimental procedures.

### Ethical considerations

Transport, housing, handling, and experimentation were performed according to the Spanish law RD53/2013 within the framework of the European Union directive on animal welfare (Directive 2010/63/EU) for the protection of animals employed for experimentation and other scientific purposes, following the guidelines for the care and welfare of cephalopods published by Fiorito et al.^15^, and the new Delegate Directive 2024/1262, with amends regarding the requirements for establishment, care, and accommodation of animals. The animal study was first reviewed and approved by the Animal Experimentation Ethics Committee of the Institute of Marine Research (IIM-CSIC). The project was subsequently evaluated and approved by the Ethics Committee of the of the Spanish National Research Council (CSIC). Finally, the procedures were authorized by the Competent Authority of Galicia (Consellería de Medio Ambiente, Territorio e Vivenda, Xunta de Galicia) under the authorization code: (ES360570202001/21/FUN.01/INM06/CGM01). All experiments conducted with these animals were performed by qualified and duly accredited personnel, and in strict accordance with the relevant institutional, national, and European guidelines and regulations. All methods were carried out following the approved protocols and in compliance with the principles of the Three Rs (Replacement, Reduction, and Refinement) to promote ethical and responsible research practices. This study was conducted and reported in accordance with the ARRIVE 2.0 guidelines to ensure transparency, reproducibility, and high standards of animal welfare.

### Preparation of cell suspensions

Octopus immune cell preparations were optimized from three sources: fresh circulating hemocytes, cryopreserved hemocytes, and white body-resident hemocytes (WBH). Cells from each source were processed according to the steps described below.

#### Preparation of fresh circulating hemocytes

A total of two adult animals were bath anesthetized by immersion in 3L of a 1.5% MgCl_2_ solution dissolved in filtered seawater (FSW) and supplemented with 1% ethanol for 5–7 min^15^. Hemolymph samples were collected from the caudal vein using a disposable syringe while the animals remained under anesthesia. The animals were returned to their aquarium, showing complete recovery from anaesthesia within 10–15 min. A maximum of 1 mL hemolymph with a cell concentration of 1–2 × 10^7^ cells/mL was extracted from each octopus, and repeated after at least 6 days, if additional hemolymph was needed for the experiments, according to Malham et al.^47^. To select the most effective antiaggregant medium, syringes were pre-filled in a 1:1 ratio with Marine Antiaggregant Solution (MAS)^27^ or Squid Ringer’s solution (SRS)^48^. Cells were observed under a light microscope at 15, 30, 60, and 120 min post-extraction to evaluate their aggregation degree and morphology. Once the optimal collection media were identified, new hemolymph samples were collected to: (i) evaluate cell viability in different maintenance cell media and (ii) analyze cell morphology and individualization degree under the microscope. For that, hemolymph was centrifuged at 300×g for 5 min at 4 °C, supernatants were gently removed, and again centrifuged at maximum speed (13,000×g) to obtain a cell-free *O. vulgaris* serum (CF-OS), which was used as medium for subsequent experiments. Cell pellets were then resuspended in a variable volume of the different media under evaluation to achieve a final concentration of 3 × 10^6^ cells/mL. The panel of maintenance media evaluated included MAS, SRS, calcium- and magnesium-free artificial seawater (CMF-ASW), Leibovitz-15 media (L-15), RPMI 1640 (Gibco), CF-OS, and PBS (alone and supplemented with 0.7 M D-mannitol). The detailed composition of all lab‑prepared media is provided in Supplementary Table 1. Cells were observed under a light microscope at 15, 30, 60, and 120 min post-extraction to assess their aggregation degree and morphology. Those media that showed the highest percentages of cell viability (see cell viability results) were further tested with the addition of 0.04% BSA (bovine serum albumin) to evaluate the effect of this supplementation on cell preservation. Cell suspensions were handled with RNase-free consumables, using 1000 µl filter tips to prevent cell clumping, and applying gentle pipetting with smooth up and down to minimize mechanical damage.

#### Preparation of cryopreserved hemocytes

Once the maintenance medium for fresh cells was selected, the same medium was used to obtain cell suspensions from cryopreserved cells. Hemocytes were thawed following the steps previously described by Costa et al.^49^. Briefly, vials were thawed in a 30 °C water bath and progressively diluted in MAS medium by alternating the addition of medium with gentle mixing movements. Cells were slowly transferred into a 50 mL tube (one drop every 5 s), and the medium was slowly and progressively diluted up to tenfold. The cells were washed twice in MAS medium by centrifugation at 300×g for 5 min at 4 °C. The final pellet was resuspended in a variable volume of selected medium until a final concentration of 3 × 10^6^ cells/mL.

#### Preparation of white body-resident hemocytes

Once the procedures involving circulating hemocytes were completed, one of the animals previously used for hemolymph collection was euthanized by immersion in 3 L of a 3% MgCl_2_ solution dissolved in FSW and supplemented with 3% ethanol for 15 min. An incision was then performed in the cephalic region, between the eyes, to mechanically destroy the brain by decapitation. The subsequent dissection of the cranium allowed access to the white body, a multilobular gland behind the eyeball located around the optic nerves, between the optic lobe and the eye orbit. The head skin, eye camera, and periorbital tissue were gently removed using fine forceps to reveal the white body. Once located, it was carefully excised using precision dissection tools and immediately preserved in MAS for further analysis. Small pieces of tissue (0.5 mm in diameter) were disaggregated following different methodologies to determine the optimal conditions: (i) mechanical disaggregation (MD); (ii) enzymatic disaggregation (ED), and (iii) combined disaggregation (CD). For MD (i), tissue samples were mechanically disrupted by gently pressing them against 100‑µm nylon meshes. Primarily disaggregated cells were passed again through sequential 100 µm + 40 µm, or sequential 100 µm + 20 µm pore sizes adding the appropriate buffer and using a drop‑by‑drop method. The resulting cell suspensions were immediately transferred into MAS medium. For ED (ii) a total volume of 960 µL MAS medium supplemented with 40 µL of collagenase IV (100 mg/mL) (Gibco) or an equivalent volume of a collagenase/dispase mixture (Merck) was added to the tissue fragments and incubated for 2 h and 30 min at 25 °C with continuous agitation (200 rpm). The CD protocol (iii) integrated both mechanical (i) and enzymatic (ii) disaggregation approaches. Briefly, white body sections were incubated with collagenase IV for 2 h and 30 min in agitation, followed by filtration through sequential 100 µm + 40 µm nylon meshes. The absence of cell debris was checked under the light microscope for all three protocols. In protocols (i) and (iii), the filtration step was repeated up to three times, depending on the purity of the resulting cell suspension.

### Cell viability quantification

Flow cytometry is considered the most accurate and sensitive method for cell viability quantification. However, it is not always feasible for routine use in all laboratories due to its operational requirements. To identify a reliable and practical alternative, two accessible and routine methodologies (automatic and visual cell counting) were evaluated in comparison with flow cytometry. In all cases, viability staining dyes that discriminate between dead and live cells were used. The LUNA-FL (Logos Biosystems) was used as an automatic cell counter, using fluorescence-based detection. For that, a total volume of 18 µL of cell suspensions was stained with 2 µL of an acridine Orange/Propidium Iodide Stain (Logos Biosystems) as cell viability staining. A volume of 10 µL of the stained sample was loaded into the slide chamber for automatic quantification. Measurements were made under optimal conditions (exposure and threshold settings for both green and red fluorescent channels) previously established. For Neubauer chamber manual counting, cells were previously stained with a 0.4% trypan blue solution in FSW in a 1:1 ratio. A volume of 10 µL of the stained suspension was loaded into the chamber and the cells were manually counted under a light microscope. For cell viability quantification by flow cytometer, a total volume of 100 µL of each cell source was incubated in the dark with 2 µL of propidium iodide (PI) (1 mg/mL) before analysis. Measurements were performed using a Cytoflex (Beckham Coulter) at the Flow Cytometry Service at CINBIO (University of Vigo).

### 10 × Chromium single-cell protocol

To establish an optimized protocol for marine cells, specifically for octopus immune cells, several steps of the 10 × Chromium single-cell workflow were analyzed and specifically modified.

#### Gel bead-in-EMulsion (GEMs) generation

In order to assess the compatibility of the selected medium with the 10 × Chromium technology, a Chromium Next GEM training kit (Ref. PN-1000143) was used prior to library preparation. This allowed testing whether the selected buffer supported the Gel beads-in-EMulsion (GEMs) generation and was therefore suitable for cell encapsulation into oil droplets. To ensure optimal compatibility with the 10 × Chromium chemistry, the EDTA concentration in the selected MAS buffer was reduced from 10 to 1 mM. This modified buffer is hereinafter referred to as MAS low. This adjustment ensured that the small proportion of MAS low contributing to the 75 µL GEM reaction resulted in a final EDTA concentration that remains below the 0.1 mM threshold specified by 10 × Genomics. Cell viability and the extent of cell aggregation at this concentration were then assessed as described in previous sections. A total volume of 31.8 µL of the Chromium training master mix (Ref. PN-220086) was mixed with the corresponding volume of cell suspension, with a cell concentration ranging from 3 × 10^6^ to 5.8 × 10^6^ cells/mL, to get a final quantity of 20,000 cells. The remaining reaction volume up to 75 µl, as recommended in the Chromium Next GEM Training kit user guide (CG000210 Rev F), was completed with nuclease-free water. Since the volume of the cell suspension within the GEMs generation reaction depends on the cell concentration obtained for the starting population, the resulting medium/water ratio could reach levels that may not bempatible with the 10 × Chromium chemistry. To this end, the ability of the modified medium to support GEMs generation was assessed, regardless of cell number, by using the maximum volume of buffer allowed by the reaction: 43.2 µL (Chromium Next GEM Training kit user guide (CG000210 Rev F)). The GEMs generation was evaluated by visual appearance of the cell suspension in the emulsion and further confirmed under a light microscope by verifying the presence of individual cells encapsulated within oil droplets.

#### Assessment of the selected medium’s impact on reverse transcription (RT) efficiency

Once the suitability of the MAS low medium for GEM generation had been established using the training kit, the next necessary step was to assess its compatibility with efficient RT. Specifically, we aimed to determine whether any of the medium’s components might interfere with a successful cDNA synthesis. Although the methodology used in the 10 × system (Chromium Next GEM Cell 3’ Kit v3.1(Dual Index) (Ref. PN-1000268)) employs a non-conventional RT methodology, in which cell lysis, RNA isolation, and RT to cDNA occur within the GEMs, our aim was to assess whether the MAS low selected buffer could potentially inhibit the reaction. To do this, 500 ng of RNA were used for reverse transcription into cDNA using the Maxima First Strand cDNA Synthesis Kit for RT-qPCR (ThermoFisher) in both reaction conditions. In the first condition, water was used to adjust the final reaction volume according to the kit instructions, while in the second, the selected medium (MAS low) was used instead to evaluate its potential impact on reaction efficiency. RNA integrity was previously checked using a Bioanalyzer (Agilent). Octopus ubiquitin expression was used as the housekeeping gene^12^ to assess the RT efficiency. Quantitative PCR (qPCR) assays were performed on a Quant Studio 3 (Applied Biosystems). A total volume of 25 μL PCR mixture included 12.5 μL of SYBR Green PCR master mix (Applied Biosystems), 0.5 μL of each primer pair 10 μM, and 1 μL of cDNA per reaction. Amplification was carried out at the standard cycling conditions of 95 °C for 10 min, followed by 40 cycles of 95 °C for 15 s and 60 °C for 1 min. Each reaction was conducted in triplicate.

### Quality control of reverse transcription (RT) and library preparation using TapeStation

Single-cell cDNA libraries were prepared following the manufacturer user guide protocol (CG000315) provided by 10 × Genomics Chromium Next GEM Single Cell 3’ Reagent Kits v3.1 (Dual Index), including Chromium Next GEM Cell 3’ Kit v3.1, 16 rxns (Ref. PN-1000268), Chromium Next GEM Chip G Single Cell Kit, 16 rxns (Ref. PN-1000127), and Dual Index Kit TT Set A, 96 rxns (Ref. PN-1000215). Following RT and cDNA amplification using the full Chromium Single Cell 3’ reagent kit (which includes the RT enzymes), an initial quality control assessment was performed to evaluate the success of reverse transcription. For this purpose, cDNA samples were analyzed using the Agilent 4200 TapeStation system with D5000 ScreenTape assays. The presence of a cDNA profile within the expected size range was used to confirm an efficient RT and amplification. Final libraries were constructed according to the 10 × Genomics guidelines (CG000315). Library integrity, fragment size distribution, and concentration were subsequently assessed using the TapeStation system, by employing a High Sensitivity HS D1000 ScreenTape device (Ref. 5067–5592) and High Sensitivity HS D1000 ScreenTape reagents (5067–5593).

### Shallow sequencing

A shallow sequencing run was conducted to evaluate the overall quality of the libraries before deep sequencing and to assess the compatibility of the selected medium. Libraries were sequenced on an Illumina S1 flow cell using a shared run with paired-end 150 bp reads (PE150), targeting approximately 5 Gb of data per sample. This low-pass sequencing approach allowed for an initial quality screening of the libraries generated for single-cell analysis. The run was configured to achieve a coverage of approximately 50,000 reads per cell, yielding a total sequencing output of ~ 3000 million reads (3000 M) across the project. The resulting data were processed using Cell Ranger v7.1.0 (10 × Genomics), employing default settings for single-cell gene expression analysis. The pipeline was used to provide key quality metrics, including the estimated number of captured cells, the mean number of reads per cell, and the median number of genes detected per cell.

### Statistics

Viability results were analyzed using a two-way ANOVA with type of medium as a fixed factor, with assumptions verified as described below. Before the analyses, normality and homogeneity of variances were checked by Shapiro–Wilk and Levene’s tests, respectively. In cases where the homogeneity of variances was violated, rank-transformed data were considered to run the ANOVA^50^. Results were considered significant at *p* ≤ 0.05. Homogeneous groups were established a posteriori with Tukey’s tests for multiple comparisons. Viability results from microscopy-based methods (LUNA-FL and Neubauer chamber) were compared with flow cytometry. Discrepancies between microscopy methods and flow cytometry were assessed by calculating absolute differences in paired viability values (LUNA-FL vs. cytometry and Neubauer chamber vs. cytometry). The mean Euclidean distance, calculated using the formula below, was used to identify which method showed the greatest deviation from flow cytometry data.

$$d = \frac{{\mathop \sum \nolimits_{i = 1}^{n} \sqrt {\left( {x_{i} - y_{i} } \right)^{2} } }}{n}$$

(where: d = distance; i = analyzed case; x = cell viability value obtained from a microscopic quantification methodology (LUNA-FL or Neubauer chamber); y = cell viability value obtained from cytometry quantification; n = number of analyzed cases). Before statistical testing, the normality of each set of absolute differences was assessed using a Shapiro–Wilk W test. Subsequently, a two-sample t-test was performed to determine whether the mean absolute differences between methods were significantly different. Results were considered significant at *p* ≤ 0.05. Statistical analyses were done using the STATISTICA v.7.0 software (StatSoft) and RStudio.

## Results

### Effect of maintenance media and dissociation methods on cell viability

**Fig. 1:**
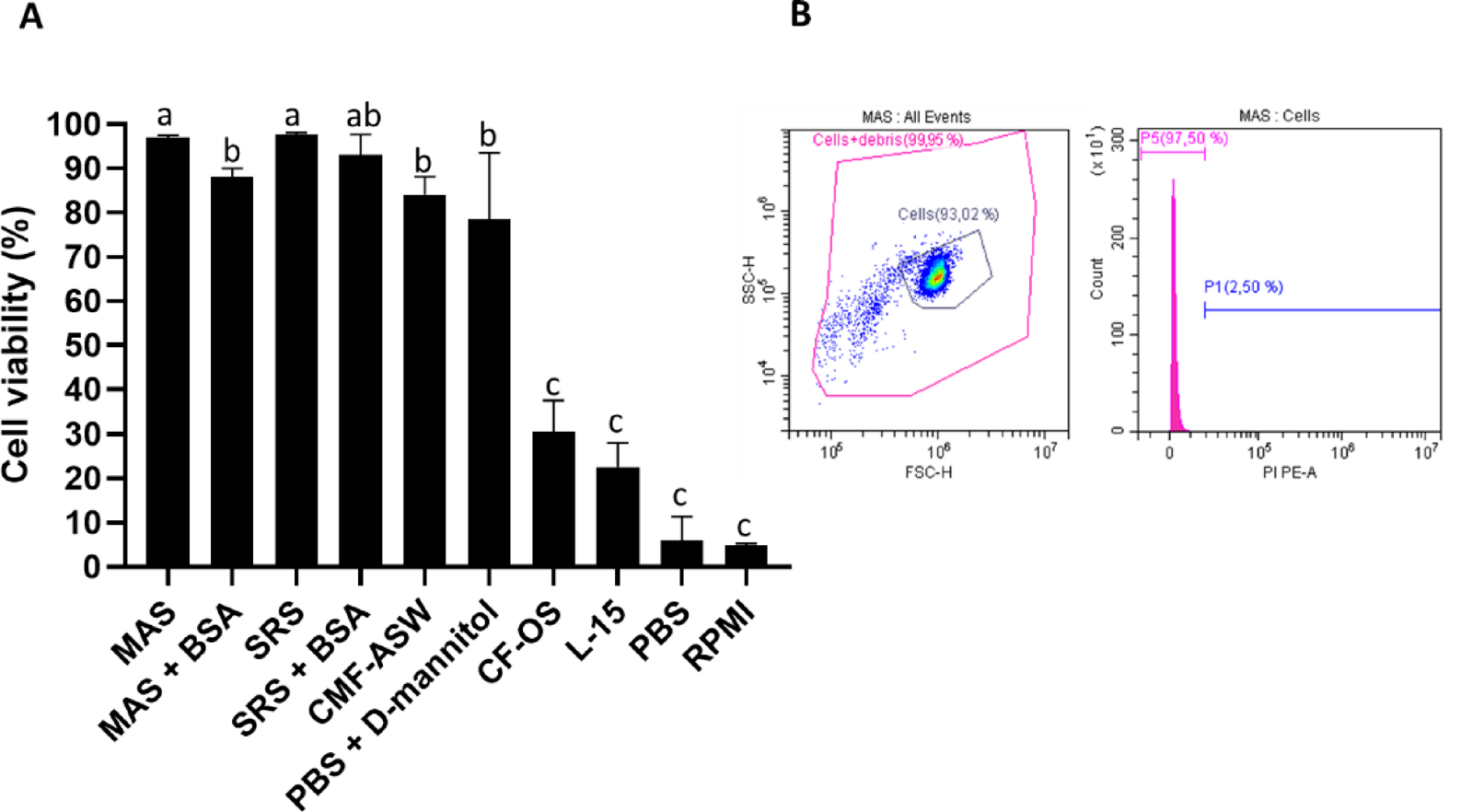
Assessment of hemocyte viability under different culture media conditions using flow cytometry. Percentage of viable hemocytes maintained in different media (**A**). Viability was assessed by propidium iodide (PI) exclusion using flow cytometry. Bars represent the mean + SEM from independent biological replicates. Different letters above bars indicate statistically significant differences between groups (*p* ≤ 0.05). Representative flow cytometry analysis of hemocytes maintained in MAS medium (the condition with the highest viability) (**B**). Dot plot showing side scatter (SSC) versus forward scatter (FSC), indicating cell size and granularity (left) and fluorescence histogram showing PI signal distribution (right). The pink curve represents the population of viable (PI-negative) cells, while the right area represents non-viable (PI-positive) cells.

**Fig. 2:**
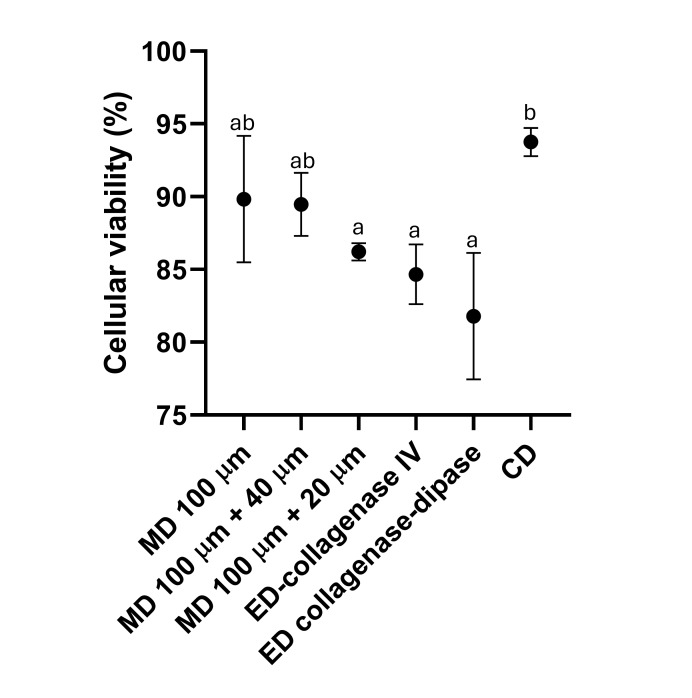
Assessment of white body cells viability using flow cytometry. Percentage of viable hemocytes obtained by different methodologies (MD: mechanical disaggregation; ED: enzymatic disaggregation; CD: combined disaggregation) with MAS used as the maintenance medium. Viability was assessed by propidium iodide (PI) exclusion using flow cytometry. Bars represent the mean + SEM from independent biological replicates. Different letters above bars indicate statistically significant differences between groups (*p* ≤ 0.05).

Among the maintenance media tested, MAS and SRS showed the highest viability of fresh circulating immune cells, reaching nearly 100% (97.05% and 97.84%, respectively) by flow cytometry (Fig. 1A). CMF-ASW and PBS with D-mannitol also maintained relatively high viability (84.05% and 78.49%), though significantly lower than MAS and SRS. In contrast, CF-OS, L-15, PBS, and RPMI exhibited the lowest rates (30.00%, 22.62%, 5.90%, and 4.88%), with no significant differences among them. Supplementation of MAS and SRS with 0.04% BSA did not improve viability; MAS even showed a significant reduction, while SRS remained unchanged. A dot plot illustrating the distribution of total cell events from a cell suspension in the optimal medium (MAS medium), along with a histogram representing PI-positive (dead) cells, is shown in Fig. 1B. For cryopreserved cells, MAS was also selected as the optimal medium based on previous results^49^. Consequently, MAS was also used for WBH suspensions. Regarding disaggregation methods (Fig. 2), mechanical approaches yielded higher viability than enzymatic ones, though differences were not significant due to high variability within treatments. The combined method achieved the highest viability and lowest variability, making it the most suitable for obtaining viable white body cell suspensions.

### Cell individualization

**Fig. 3:**
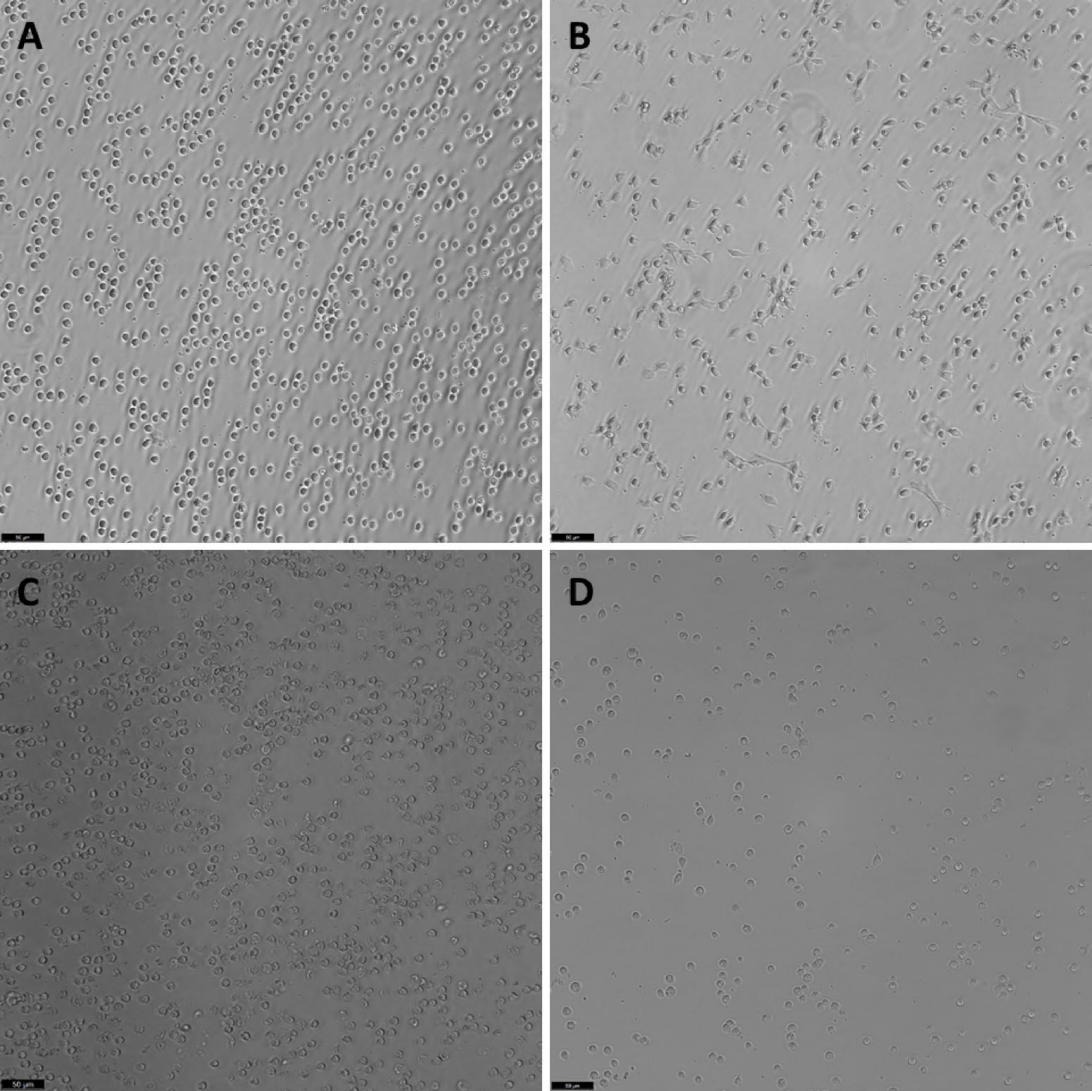
Morphology of hemocytes observed under light microscopy. Fresh cells maintained in MAS medium (**A**), fresh cells maintained in SRS medium (**B**), cryopreserved and thawed cells maintained in MAS medium (**C**), and cells from the white body obtained after using the combined disaggregation method and maintained in MAS medium (**D**). All images were taken after 2 h of incubation under the respective conditions. Scale bar: 50 µm.

In addition to viability, cell individualization is also a crucial requirement for single-cell RNA sequencing assays. Light microscope observations over two hours revealed that cells maintained in MAS medium exhibited the highest levels of individualization, with no evidence of cell aggregates for both fresh circulating hemocytes (Fig. 3A) or cryopreserved hemocytes (Fig. 3C). WBH were also properly individualized in MAS medium, although exhibiting minimal debris (Fig. 3D). In contrast, although SRS medium also exhibited high cell viability, it induced cell interactions through the formation of pseudopodia, hindering the individualization of cells (Fig. 3B). Additional images showing cell morphology in the remaining tested media, as well as at all the analyzed time points (0, 30 min, 1 h, and 2 h), are provided in Supplementary Fig. 1.

### Assessing routine cell counting methods vs flow cytometry standards

**Fig. 4:**
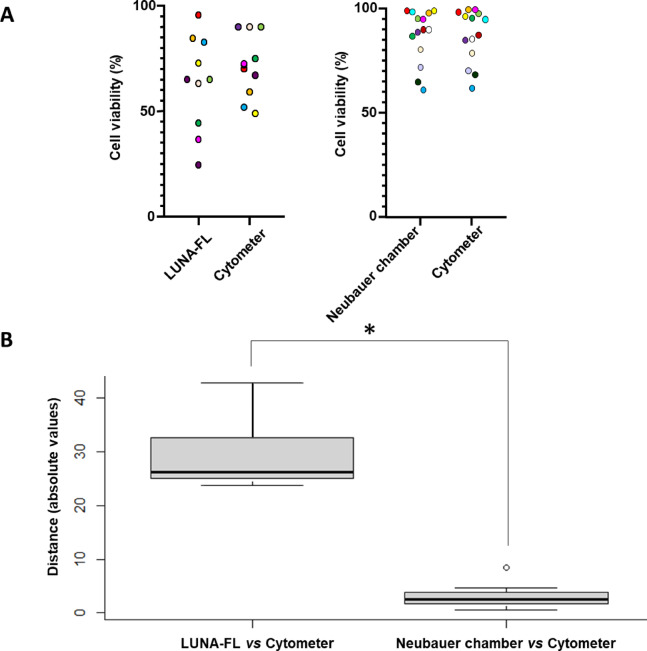
Pairwise comparison of cell viability measurements between methods. Viability of octopus hemocytes was quantified using flow cytometry (propidium iodide staining); LUNA-FL automated cell counter (acridine orange staining) with the following acquisition parameters: cell size preset 3_60, green/red thresholds 5/5, green/red exposure 6/11); and Neubauer chamber (trypan blue staining). Two comparisons are shown: LUNA-FL vs. flow cytometry, and Neubauer vs. flow cytometry. Each point represents a single sample measured by both methods, with matching colors indicating the same sample across comparisons (**A**). Comparison of absolute distances between cell viability measurements obtained by different microscopy methods vs. flow cytometry (**B**). Each boxplot represents the interquartile range (IQR), which covers from the first quartile (Q1, 25%) to the third quartile (Q3, 75%). The horizontal line inside each box indicates the median (Q2, 50%). The vertical lines (whiskers) extend to the smallest and largest values within 1.5 times the IQR from the lower and upper quartiles, respectively. Points beyond the whiskers are plotted individually as potential outliers. Statistical differences between groups are indicated by an asterisk (*).

Flow cytometry is considered the reference method for assessing cell viability. To identify the most comparable routine approach, hemocyte counts from LUNA-FL and the Neubauer chamber were compared to flow cytometry (Fig. 4). The Euclidean distance between LUNA-FL and flow cytometry was 29.36, whereas the Neubauer chamber showed a much lower distance of 3.03 (Table 1). A two-sample t-test confirmed this difference as statistically significant (*p* ≤ 0.001), indicating that Neubauer counts are substantially closer to flow cytometry than LUNA-FL. As flow cytometry was used as the reference for cell counting due to its objectivity and sensitivity, a greater distance from its results indicates lower accuracy. According to these results, the proposed routine quantification method was cell counting using a Neubauer chamber.

**Table 1:** Comparison of cell viability measurements using different methods.

Sample	Comparison 1			Sample	Comparison 2		
	% of cell viability		Sqr (dif^2^)		% of cell viability		Sqr (dif^2^)
	LUNA-FL	Flow cytometry			Neubauer	Flow cytometry	
1	95.7	70.1	25.6	11	95.2	97.8	2.60
2	44.3	75.2	30.9	12	87	95.5	8.5
3	84.5	59.6	24.9	13	98	99.8	1.80
4	63.1	90	26.9	14	88.9	85.1	3.80
5	65	90.1	25.1	15	95	99.6	4.60
6	24.5	67.3	42.8	16	99	98.5	0.50
7	72.9	49.2	23.7	17	98.51	94.9	3.61
8	82.8	50.1	32.7	18	99	96.4	2.60
9	36.6	72.3	35.7	19	90	87.6	2.40
10	65	90.3	25.3	20	90	85.4	4.60
		**Distance**	**29.36**	21	72	70.3	1.70
				22	65	68.4	3.40
				23	61.2	62.0	0.80
				24	80.5	78.9	1.60
						**Distance**	**3.03**

Comparison 1: LUNA-FL vs. Flow Cytometry. Comparison 2: Neubauer vs. Flow Cytometry. Values in bold indicate the Euclidean distance calculated from the squared diff erences (dif^2^)between methods.

### GEMs generation

**Fig. 5:**
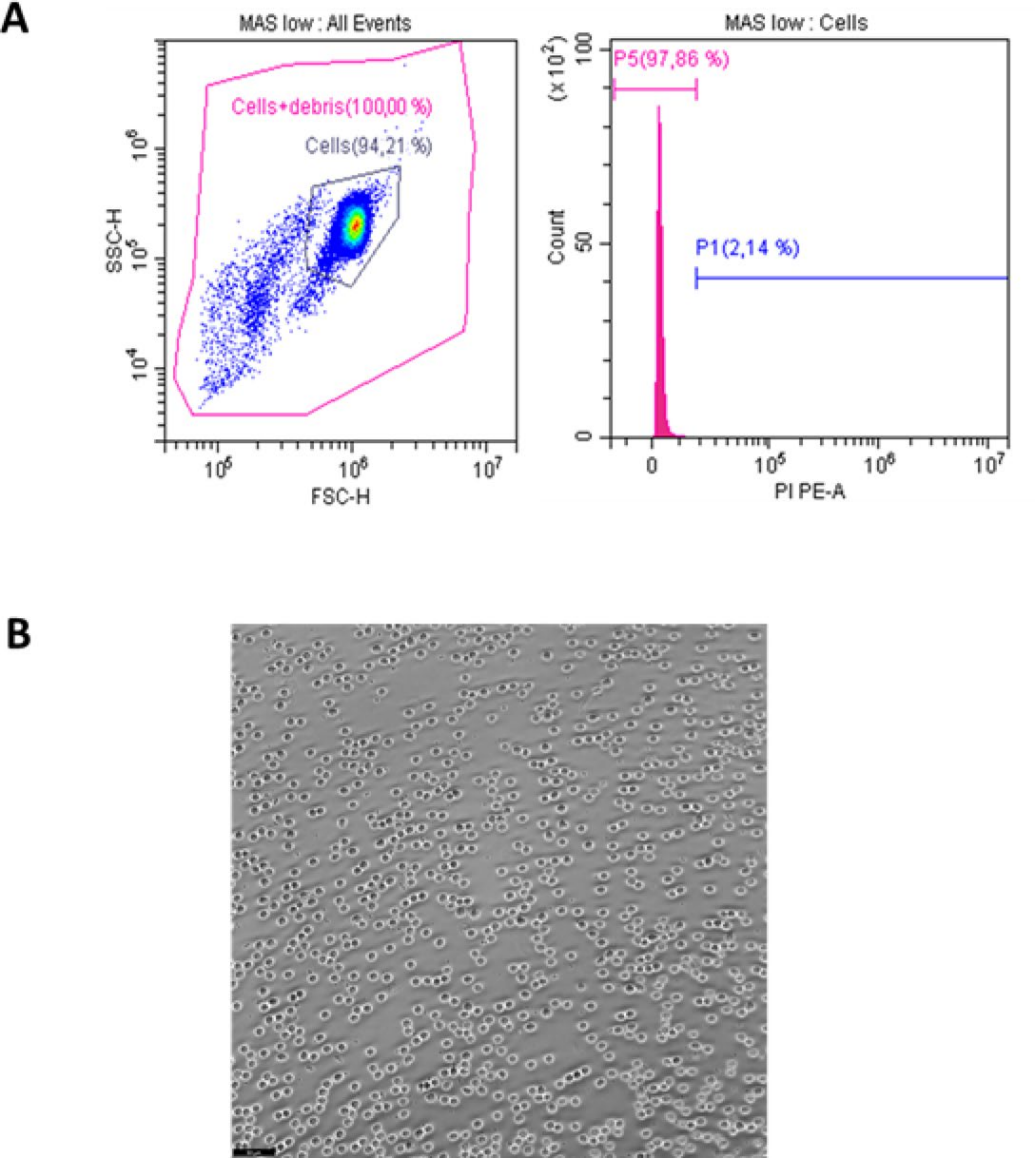
Viability and morphology of hemocytes maintained in MAS low medium. Dot plot showing side scatter (SSC) versus forward scatter (FSC), indicating cell granularity and size (left), and fluorescence histogram showing propidium iodide (PI) fluorescence intensity (right). The left peak corresponds to viable (PI-negative) cells, while the right area represents non-viable (PI-positive) cells (**A**). Light microscopy image showing the morphology of hemocytes maintained in MAS low medium for 2 h. Scale bar: 50 µm (**B**).

**Fig. 6:**
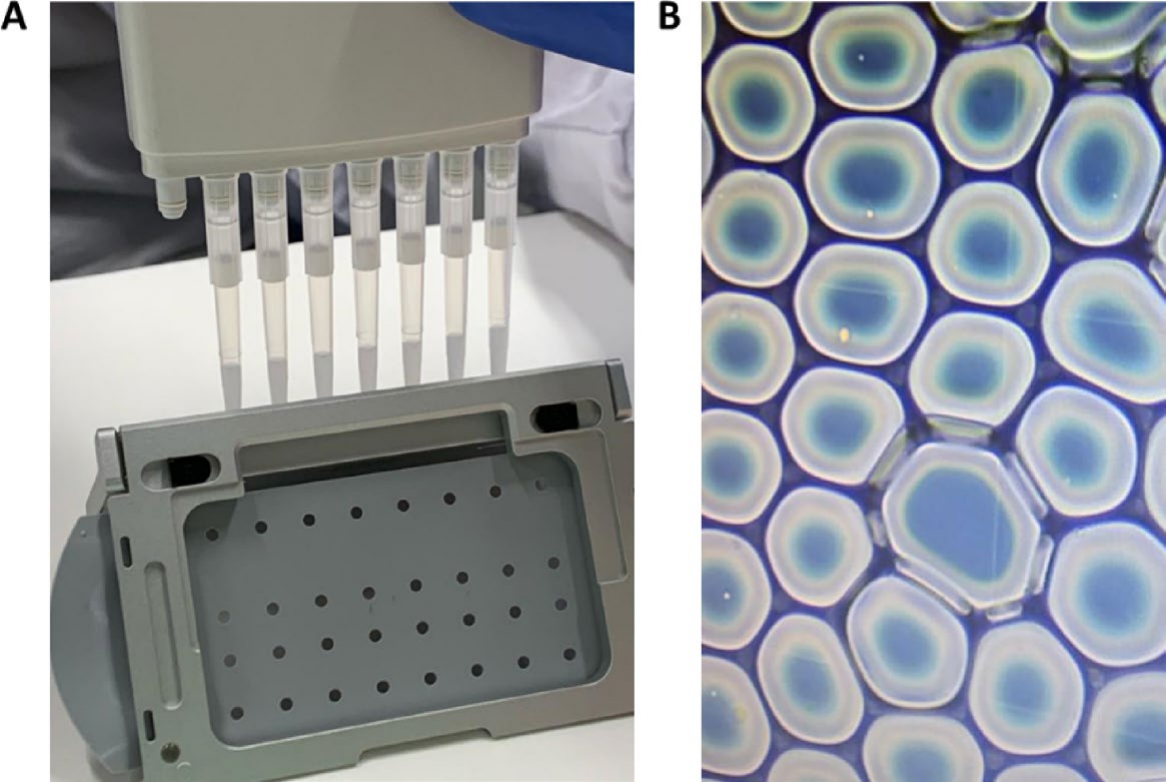
Assessment of GEM generation in the presence of the MAS low medium using a 10 × Chromium training kit. Representative image showing the characteristic white emulsion collected in pipette tips after GEM generation, indicating successful droplet formation for all samples (**A**). Light microscopy image showing individual cells encapsulated within GEMs, confirming proper emulsification and cell compartmentalization in the MAS low medium (**B**).

To evaluate the compatibility of MAS low medium with GEM generation, cells were resuspended in this formulation with reduced EDTA for optimal performance with 10 × Chromium chemistry. Before GEM generation, flow cytometry analysis confirmed viability percentages comparable to MAS (Fig. 5A), and microscopy showed similar morphology and proper individualization without aggregation (Fig. 5B). GEM formation was verified by the characteristic white precipitate in pipette tips (Fig. 6A) and direct visualization of droplets containing single cells (Fig. 6B). These results demonstrate that MAS low supports correct GEM encapsulation and meets quality control requirements for single-cell sample preparation.

### RNA integrity and reverse transcription (RT) efficiency

**Fig. 7:**
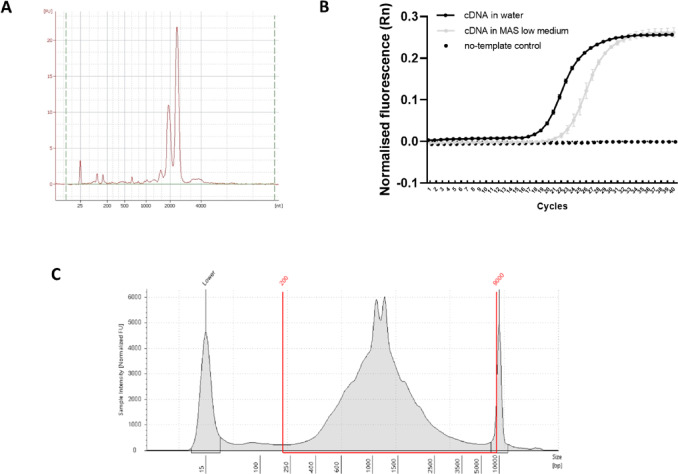
Evaluation of reverse transcription efficiency and amplified cDNA library quality using MAS low medium. RNA integrity number (RIN) values assessed by Agilent Bioanalyzer for RNA samples prior to reverse transcription (**A**). qPCR amplification curves of the octopus ubiquitin gene from cDNA synthesized using either water (control) or MAS low buffer in the reverse transcription reaction. A no-template control (NTC) was also included (**B**). Electropherogram profiles of cDNA analyzed by Agilent 4200 TapeStation system with High Sensitivity D5000 ScreenTape assays (**C**).

We assessed whether MAS low medium interfered with both RNA integrity and RT efficiency. Bioanalyzer profiles showed the characteristic cephalopod double-peak pattern^51^, confirming high RNA quality and no degradation (Fig. 7A). RT efficiency was also tested by comparing RNA diluted in MAS low (mimicking the conditions inside the GEMs) versus RNA diluted in water as a positive control. Taking into account that the MAS low volume within each GEM represents only a fraction of the total reaction volume (corresponding to 20,000 cells collected for the 10 × Genomics workflow), and the remaining volume was water, we aimed to simulate the worst-case scenario by performing the reaction with RNA fully diluted in MAS low and assessing the outcome. qPCR amplification of ubiquitin was successful in both conditions, with only a ~ 2-cycle Ct increase for MAS low (Ct = 22.4 vs. 20.2), indicating compatibility (Fig. 7B). TapeStation analysis after RT following the 10 × protocol revealed the expected cDNA size range (250–5000 bp) and yield, with no signs of degradation (Fig. 7C). These results confirm that MAS low does not compromise RNA integrity or RT performance for library preparation.

### Library quality control and shallow sequencing

**Fig. 8:**
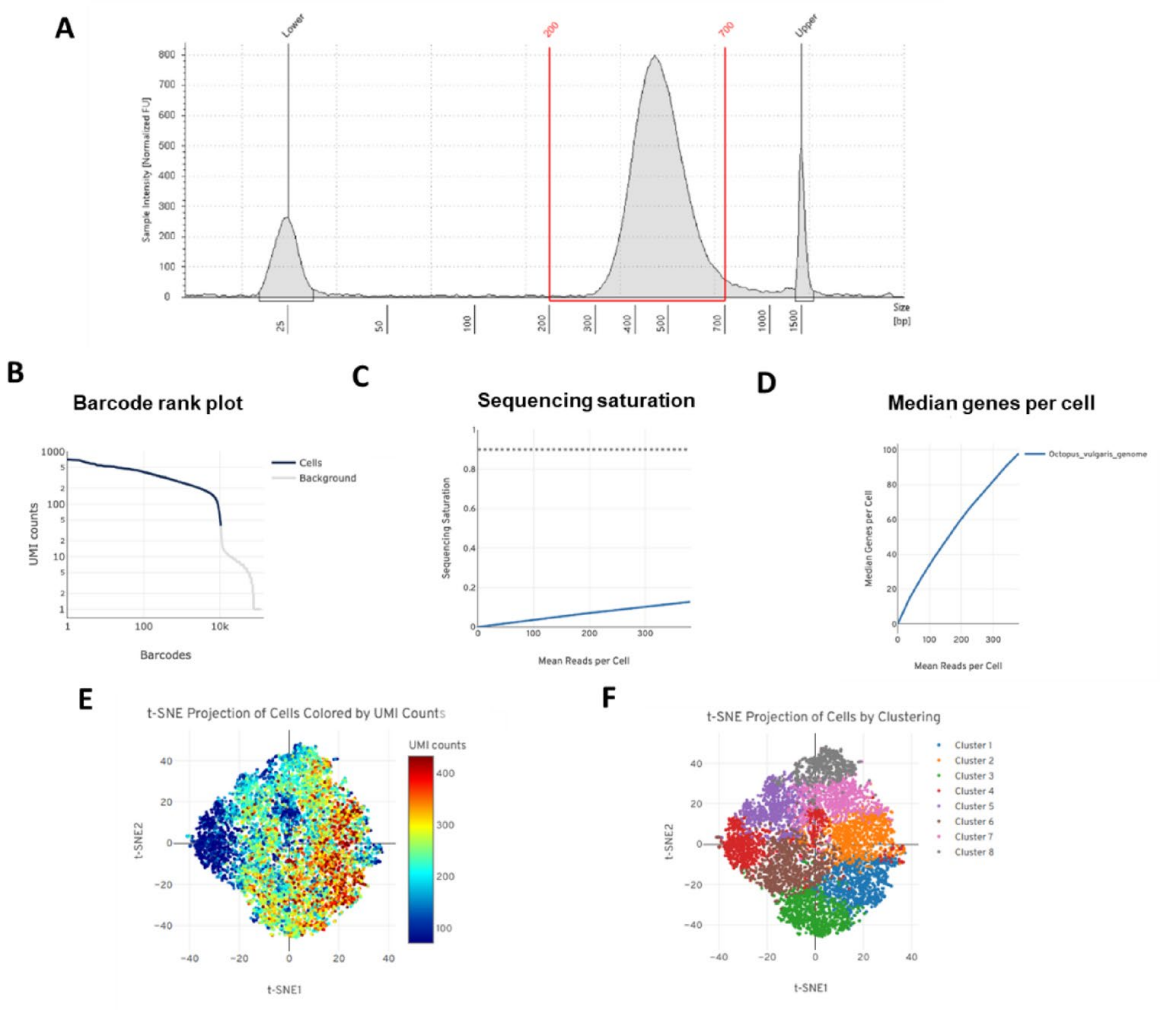
Quality assessment and analysis of the single-cell 3’gene expression library and sequencing data. TapeStation electropherogram showing the size distribution of the cDNA library (**A**). Barcode rank plot depicting the distribution of Unique Molecular Identifiers (UMIs) per barcode (**B**). Sequencing saturation curve showing low saturation values and an increasing trend of mean reads per cell with sequencing depth (**C**). Relationship between median genes detected per cell and downsampled sequencing depth (mean reads per cell) (**D**). t-SNE plot displaying the heterogeneous distribution of UMIs per cell across the dataset (**E**). t-SNE clustering illustrating distinct cell subpopulations identified within the sample, reflecting transcriptomic diversity captured by the library (**F**).

A barcoded single-cell 3′ Gene Expression library was prepared following the 10 × Genomics protocol using 25% of the cDNA yield. TapeStation analysis showed a well-defined and homogeneous electropherogram with a central peak between 200 and 700 bp, confirming proper library integrity and absence of degradation (Fig. 8A). After shallow sequencing, the distribution of the barcodes based on the number of Unique Molecular Identifiers (UMIs) showed a clear difference between the presence of real cells in the sequencing and the background noise. The curve shows a sharp drop in background signal, indicating a clear threshold for cell detection (Fig. 8B). A positive correlation between reads per cell and sequencing saturation (Fig. 8C) indicates that deeper sequencing would improve gene detection. Median gene counts per cell increased with sequencing depth (Fig. 8D). The t-SNE (t-distributed Stochastic Neighbor Embedding) analysis displayed heterogeneous UMI distribution per cell (Fig. 8E) and well-defined clusters (Fig. 8F), suggesting, as a preliminary result, the existence of distinct cell subpopulations in the sample. The clustering and broad UMI distribution confirm successful library construction and ensure a diverse, representative transcriptomic profile.

## Discussion

Single-cell transcriptomics has revolutionized the understanding of cell diversity, enabling unprecedented insights into gene expression at the individual cell level^52^. This powerful technique has transformed a wide range of fields, extending from developmental biology to immunology, allowing researchers to dissect complex cell landscapes with remarkable resolution^28^. This impact is also evident in marine invertebrates, where recent single-cell studies have uncovered diverse neural cell types in cephalopods and revealed heterogeneous immune cell populations in molluscs and crustaceans^41–45^. By overcoming the limitations of bulk RNA sequencing, single-cell approaches provide a more detailed and specific view of heterogeneous or poorly characterized cell populations, revealing rare or novel cell types and dynamic transcriptional states that would otherwise remain unknown. Among its many applications, the study of immune cell populations has emerged as one of the most promising and technically demanding applications of single-cell transcriptomics. This is particularly relevant in invertebrates, which lack an adaptive immune system, and immune cells cover different functions. Traditionally, immune cells have been studied and classified based on morphological characteristics and bulk functional immune assays, such as phagocytic ability, ROS production, lysozyme activity, or transcriptomics analysis, which have provided valuable insights into immune function^18,53^. However, these population-level approaches overlook the heterogeneity at the individual level. In contrast, single-cell transcriptomics enables a much deeper resolution, revealing functional diversity and cell specialization that would be undetectable using conventional methods^54^.

Despite these advantages, single-cell methodologies present significant challenges. Factors such as sample preparation, cell viability, RNA integrity, and species-specific biases can greatly impact the quality and reliability of the data^28,33,54^. These issues become even more complex when working with marine organisms. The vast diversity of marine taxa, in terms of morphology and fundamental biological traits such as feeding and reproductive strategies, coincides with remarkable cell complexity. These difficulties are even more pronounced in non-model marine species, which frequently lack established molecular tools and reference datasets. Additional levels of complexity arise from their unique cell features, osmotic sensitivity, and poikilothermic nature, all of which require tight control of environmental conditions during sample handling to preserve cell viability and prevent stress-induced artifacts. For instance, the osmotic conditions of marine invertebrates differ significantly from those of standardized models, requiring specific adaptations to experimental protocols in order to maintain cell integrity and RNA quality. Moreover, immune cells in many invertebrates lack a coagulation system but exhibit a strong tendency to aggregate in response to stimuli, an intrinsic feature of their immune behaviour^56,57^. While this response is biologically meaningful, it complicates the cell isolation for single-cell transcriptomic analysis, affecting cell individualization and compromising data quality.

A complete characterization of the cephalopod immune system requires the analysis of both circulating hemocytes and tissue-resident cells from the white body. However, the isolation of these two cell types involves different technical challenges due to the differences in their biological and handling requirements. In light of this, a dual optimization strategy was undertaken for *O. vulgaris* immune cells, aimed at enabling reliable single-cell RNA sequencing.

Previous studies have reported several types of cells in the hemolymph of this species, although a consensus regarding their classification and functional identity is not fully clear. Troncone et al.^27^ described up to three distinct hemocyte types based on morphological, functional, and flow cytometric analyses. In contrast, Castellanos-Martínez et al.^16^ identified only two main populations. Both studies, published at similar times, identified two main hemocyte populations. Troncone et al.^27^, however, reported an additional group which may represent a subpopulation of one of the two primary types rather than a distinct class. Novoa et al.^26^, using electron microscopy, also reported only two hemocyte types in both peripheral hemolymph and the white body, suggesting that one type differentiates from a less developed precursor. Therefore, it still remains unclear whether cephalopod hemocytes represent truly distinct cell types or transitional stages. Bulk RNA-sequencing studies have provided valuable insights regarding gene expression^16^, but uncertainties remain regarding the precise number, identity, and classification of cephalopod immune cell types. This lack of resolution highlights the limitations of conventional approaches and reinforces the need for high-throughput, single-cell transcriptome-based methods.

Several single-cell studies on immune marine cells have revealed their heterogeneity and functional specialization^44,58,59^. In cephalopods, this approach is key to resolving questions about hemocyte diversity, lineage origins, and whether differentiation occurs in the white body or bloodstream. However, platforms require > 90% viability and proper cell individualization. Although various buffers have been tested, previous works lack stepwise workflows, systematic quality controls, and assessments of compatibility with 10 × Genomics chemistry. Our protocol addressed these gaps, optimizing viability, dissociation, RT, and library construction. PBS, a widely used medium in standardized single-cell protocols and recommended by the 10 × Genomics platform^60^, and RPMI 1640, a common mammalian cell culture medium, both failed to maintain hemocyte viability in our experiments. These media are isotonic solutions (~ 280–300 mOsm/L), formulated to match mammalian cells, but this is far below the osmolality of octopus hemocytes (~ 1170 mOsm/kg H₂O)^61^. This mismatch likely causes osmotic stress, water influx, membrane rupture, and cell lysis. Accordingly, hemocyte viability was very low in PBS or RPMI, confirming that osmolality is critical for cell integrity. Adding D-mannitol to PBS, an osmotic agent used clinically and in electroporation protocols^62^, improved viability. Scully and Klein^63^ reported similar benefits in *Ciona intestinalis* blood cells. However, 0.7 M D-mannitol raised osmolality only to ~ 700 mOsm/L, still below optimal for octopus hemocytes, explaining why viability improved compared to PBS alone but remained lower than optimized buffers. Scully and Klein^63^ also tested CMF-ASW, achieving ~ 50% survival, similar to PBS + D-mannitol. In contrast, Styfhals et al.^42^ reported ~ 85% viability for octopus brain cells in CMF-FSW, which is compositionally similar to CMF-ASW. Their medium included 0.04% BSA, likely contributing to higher viability. In our study, CMF-ASW (without BSA) yielded lower viability than MAS or SRS, which reached nearly 100%. These findings led us to focus subsequent experiments on those media. Differences in handling, buffer composition, cell type, or species-specific requirements may explain discrepancies between studies, including those by Scully and Klein^63^.

L-15 medium has been widely used in primary cultures of several fish species^64,65^, and it has also been referenced in diverse studies involving cephalopods. For example, Songco-Casey et al.^41^ used L-15 in optic lobe cell suspensions from *O. bimaculoides* to perform cDNA libraries for scRNA-seq. Similarly, Styfhals et al.^42^ employed L-15 medium to resuspend brain cells from *O. vulgaris*, supplementing it with a cocktail of different salts. On the other hand, Masselli et al.^66^ supplemented the L-15 medium with 10% hemolymph, which supported cell viability for up to 24 h in primary neuron cultures of *O. vulgaris*. In contrast, our experiments with octopus hemocytes showed that the standard formulation of L-15 did not effectively support cell survival, yielding low viability levels comparable to those observed in PBS and RPMI. Despite its widespread use in other aquatic species and cephalopod cell types, unmodified L-15 medium appears unsuitable for maintaining hemocyte viability in cephalopods. Surprisingly, hemocytes suspended in their own hemolymph showed viability below 30%. This reduction may result from oxidative modification of hemocyanin upon exposure to atmospheric oxygen. Preliminary data by Zheng et al.^67^ indicate that hemocyanin not only transports oxygen but also helps maintain redox homeostasis. Excessive oxidation outside the organism likely disrupts this function, causing redox imbalance and increased reactive oxygen species (ROS). Combined with pH fluctuations and proteolytic activity, this pro-oxidant environment may create cytotoxic conditions ex vivo, making native hemolymph suboptimal for hemocyte viability. Single-cell approaches have been applied to hemocytes from various marine invertebrates^43–45^, but most studies lack details on media or buffers used during isolation. Key aspects such as viability, number of viable cells, and conditions for maintaining integrity are often not reported, limiting reproducibility and cross-species applicability. Our study provides a standardized evaluation of different media, including quantitative viability data, to support optimized protocols for single-cell applications in marine invertebrates. MAS and SRS media maintained high viability and have been described as anti-aggregation solutions in octopus and other cephalopods^48,49^. However, notable behavioral differences were observed: in SRS, cells showed active morphologies with pseudopodia and cell–cell interactions, which are critical for single-cell encapsulation. This behavior, typical of responsive cells like macrophages, is likely driven by ionic composition of SRS, especially Mg^2+^ and Ca^2+^, which promote cytoskeletal rearrangement and adhesion in other molluscs^69^. In contrast, MAS medium, composed mainly of glucose, citric acid, and trisodium citrate, maintained high viability while preventing cell–cell interactions. Citrate chelating properties likely reduce adhesion by sequestering divalent cations^71^, making MAS more suitable for applications requiring cell individuality, such as microencapsulation or single-cell analysis. Moreover, MAS meets the requirements of the 10 × Genomics Chromium platform, which recommends calcium-free conditions and magnesium concentrations below 3 mM to ensure optimal single-cell partitioning and minimize aggregation^60^. BSA was tested as a supplement to MAS and SRS, the two most effective media, to evaluate its potential to improve viability and reduce aggregation. This supplementation is common in single-cell workflows, including those recommended by 10 × Genomics, as it stabilizes cells, prevents adhesion to plastic, and creates a more defined microenvironment^60,72^. However, in our experiments, 0.04% BSA did not improve viability or morphology. This suggests that MAS and SRS already provide sufficient stabilization for octopus hemocytes, making BSA unnecessary under these conditions. Glucose and citrate in MAS and the ionic balance in SRS may confer physicochemical properties that support membrane stability and reduce mechanical stress, explaining the lack of additive effect. Our findings agree with Meng et al.^44^, who applied single-cell sequencing to oyster hemocytes using a medium similar to MAS. These results reinforce the broader applicability of MAS across marine invertebrates. However, Meng et al.^44^ did not report detailed validation steps such as viability, dissociation efficiency, RT success, or library quality. The optimized pipeline we propose provides a validated framework that strengthens confidence in the data and facilitates application to other species.

The tissue dissociation method significantly influences the quality, viability, and reliability of single-cell preparations, especially in non-model organisms. Mechanical techniques such as homogenization and filtration are generally gentler, preserving membranes and morphology^73^, but they are less effective for tissues with dense extracellular matrix^74^. Enzymatic methods improve tissue breakdown and cell release but require careful optimization of digestion time and enzyme concentration to avoid viability loss^75^. Combined mechanical and enzymatic approaches have been proposed to enhance efficiency while minimizing drawbacks^76^. Our results in white body tissue confirm that this strategy offers the best balance between dissociation efficiency and viability. In marine invertebrates, and particularly cephalopods, the absence of standardized protocols and tissue-specific properties highlight the need for tailored dissociation strategies, especially for downstream applications that require preserving cell individuality, such as single-cell encapsulation or expression analysis.

Developing optimized protocols for marine organisms, both for tissue dissociation and handling of circulating cells, has often limited the work with fresh cells. As a result, many researchers use single-nucleus RNA sequencing (snRNA-seq), which avoids the need for whole-cell viability but loses cytoplasmic transcripts, reducing detection of genes with low nuclear localization^77^. Although nuclear profiling has provided insights into marine organisms^78,79^, it often misses aspects of cell state, functional diversity, and post-transcriptional regulation. Cryopreservation offers an alternative that preserves full transcriptomic information. Using a protocol previously optimized for octopus hemocytes^49^, single-cell RNA sequencing can now be performed on whole cells. MAS medium supplemented with 15% ethylene glycol enabled cryopreservation at −80 °C for 15 weeks with high viability, providing a reliable option when fresh cells are not available. These findings highlight the need for robust, species-specific protocols for isolation, preservation, and individualization of whole cells to fully exploit single-cell technologies in cephalopods and other marine molluscs.

Although LUNA-FL automated cell counter is a widely standardized method recommended by platforms such as 10 × Genomics, our results showed lower correlation with flow cytometry compared to manual Neubauer counts. This may be due to LUNA-FL algorithms being optimized for mammalian cells processed in isotonic solutions like PBS^80^, whereas octopus hemocytes require media compatible with marine physiology. MAS medium contains high salt concentrations to match physiological osmolality, which may increase refractive index and reduce optical clarity, interfering with LUNA-FL autofocus and image segmentation. These optical changes can lead to inaccurate counts in marine samples^81^. Therefore, flow cytometry and Neubauer chamber counts are more reliable for viability assessment under these conditions.

Beyond ensuring high viability, the selected medium must also be compatible with platform chemistry. Since MAS was designed to maintain hemocyte viability under physiological conditions, we evaluated its impact on single-cell encapsulation efficiency and downstream reactions, an essential step to validate its suitability for high-throughput single-cell transcriptomics workflows.

The successful generation of GEMs with MAS low shows that, despite its high salinity and marine-compatible formulation, the buffer does not interfere with droplet formation or emulsification chemistry. This suggests that MAS low composition, particularly its salinity, including glucose and chelating agents, remains compatible with 10 × Chromium operational parameters. While standard MAS was initially used to prevent hemocyte aggregation, a modified version (MAS low) was later formulated to reduce EDTA from 10 to 1 mM to meet 10 × Genomics recommendations (Ca^2+^-free, < 3 mM Mg^2+^, and 0.1 mM EDTA) and minimize enzymatic inhibition^60^. MAS low preserved cell separation and viability (> 90%) after medium exchange. To rigorously assess the buffer’s compatibility, we simulated the worst-case testing with maximum buffer volume raised EDTA to ~ 0.5 mM, above the recommended limit (0.1 mM), yet GEM integrity and single-cell encapsulation were unaffected. These results confirm MAS low compatibility with early 10 × workflow steps and support its use for marine invertebrate cells. However, downstream reactions such as RT depend on divalent cations (e.g., Mg^2+^) as cofactors. Lower Mg^2+^ may reduce RNase H activity, increasing RNA stability but potentially impairing enzymatic steps in library preparation^82^. Therefore, further validation was required to ensure compatibility with later stages.

Although the RT step is a critical part of the 10 × Genomics single-cell RNA-seq workflow, the exact chemical composition and enzymatic formulation within the GEMs remain proprietary and are not publicly disclosed. Thus, it is not possible to fully replicate the RT conditions used by 10 × Genomics outside their system. To explore whether the EDTA concentration present in the MAS low buffer could impair RT, we evaluated an additional hypothetical worst-case scenario in which the maximum allowable volume of MAS low was used, yielding the highest possible EDTA concentration in the reaction. To evaluate potential inhibitory effects, we performed in vitro RT reactions using MAS low buffer. A slight delay in the amplification of the housekeeping gene was observed, as indicated by a shift in the threshold cycle (Ct). Despite this moderate impact on initial enzymatic efficiency and slower reaction kinetics, the RT reaction reached a plateau, indicating that the process proceeded to completion. Importantly, this shift was only observed under these intentionally extreme conditions and did not affect cDNA synthesis or library preparation under the actual 10 × workflow, where MAS low contributes only minimal carry-over. RNA integrity and fragment profiles analysis confirmed high-quality material and the characteristic cephalopod RNA double peak pattern^51^. Such findings align with previous reports indicating that minor RT efficiency reductions, reflected by small Ct shifts, did not affect library quality or sequencing outcomes^83^. cDNA and sequencing quality metrics are consistent with established benchmarks in single-cell transcriptomics, preserving fragment integrity and transcript diversity, which is essential for accurate representation in non-model species^84^. Although sequencing saturation remained relatively low (an expected outcome at moderate sequencing depths, such as shallow sequencing), this does not compromise the interpretability of the dataset. On the contrary, shallow sequencing was intentionally employed as a quality control step rather than for exhaustive transcriptome capture. Continued increase in gene detection with sequencing depth confirms that transcriptomic coverage was not yet saturated^85,86^. This approach is suitable for exploratory studies focused on cell diversity and complexity. Identification of distinct transcriptional clusters and a broad distribution of UMI counts per cell demonstrated biological heterogeneity, supporting successful dissociation and library preparation. These features are critical in studies aiming to identify distinct cell populations, especially in species with limited genomic resources or undefined cell markers. Previous work has highlighted that cluster separation and transcriptional heterogeneity are strong indicators of successful capture and minimal technical bias^87^. The absence of degradation signals suggests that media and handling preserved RNA stability and cell integrity. This aligns with prior studies emphasizing the impact of ionic composition and chelating agents on cell physiology and transcript preservation^88^. In this context, pre-analytical parameters, such as buffer formulation, temperature control, and timing, emerge as critical elements for preserving the biological signal in marine invertebrate scRNA-seq experiments. Overall, these results show that shallow sequencing was not only useful as a quality control step but also provided valuable insights into sample complexity and cell-type diversity. Despite technical challenges, this work confirms the feasibility of applying single-cell transcriptomics to non-traditional models and underscores key considerations for expanding transcriptomic profiling to marine organisms.

In summary, this study delivers a validated protocol for single-cell transcriptomic analysis of *O. vulgaris* hemocytes, covering critical pre-analytical steps such as cell isolation, viability preservation, tissue dissociation, and accurate quantification. Unlike previous approaches that lack stepwise validation, our workflow standardizes and optimizes each stage, ensuring reliable representation of biological samples and full compatibility with the 10 × Genomics platform. Shallow sequencing confirmed distinct hemocyte populations, supporting transcriptomic diversity preservation. This pipeline opens the door to applying single-cell technologies to marine invertebrate immune cells, providing a valuable resource for advancing marine biology, understanding cephalopod immunity, and promoting health monitoring and sustainable aquaculture.

## Supplementary Information

Below is the link to the electronic supplementary material.

Supplementary Material 1

Supplementary Material 2

## Data Availability

The single-cell RNA raw sequencing data (shallow sequencing) generated in this study have been deposited in the NCBI Sequence Read Archive (SRA) under BioProject accession number PRJNA1376517 (runs SRR36345357). https://dataview.ncbi.nlm.nih.gov/object/PRJNA1376517?reviewer=cchhm9grrv566vc35vi0tr006j.
