# Supplementary material for: Comprehensive guide for optimizing octopus immune cell preparation to enhance single cell RNA sequencing success
Source: Sci Rep. 2026 Apr 10;16:16855. doi: 10.1038/s41598-026-47700-6 (PMC13230822; doi:10.1038/s41598-026-47700-6)
Supplement: Supplementary file 2 — Supplementary Material 2 [file 41598_2026_47700_MOESM2_ESM.docx]

**Supplementary Table 1.** Detailed composition and molar concentrations of the media and solutions used in the study.

| **Medium** | **Reactive** | **Final concentration** |
| --- | --- | --- |
| Marine Antiaggregant Solution (MAS)  (Troncone et al., 2015) ^27^ | Glucose | 0.1 M |
|  | Trisodium citrate • 2 H_2_O | 15 mM |
|  | Citric Acid • 1 H_2_O | 13 mM |
|  | EDTA | 10 mM |
|  | NaCl | 0.45 M |
| MAS-low | Glucose | 0.1 M |
|  | Trisodium citrate • 2 H_2_O | 15 mM |
|  | Citric Acid • 1 H_2_O | 13 mM |
|  | EDTA | 1 mM |
|  | NaCl | 0.45 M |
| MAS + BSA | Glucose | 0.1 M |
|  | Trisodium citrate • 2 H_2_O | 15 mM |
|  | Citric Acid • 1 H_2_O | 13 mM |
|  | EDTA | 10 mM |
|  | NaCl | 0.45 M |
|  | BSA | 0.04 % |
| Squid Ringer’s solution (SRS)  (Nyholm et al., 2009) ^48^ | Magnesium Chloride • 6H_2_O | 25 mM |
|  | Calcium chloride • 3 ½ H_2_O | 10 mM |
|  | Potassium chloride | 10 mM |
|  | Sodium chloride | 530 mM |
|  | Hepes | 10 mM |
| SRS + BSA | Magnesium Chloride • 6H_2_O | 25 mM |
|  | Calcium chloride • 3 ½ H_2_O | 10 mM |
|  | Potassium chloride | 10 mM |
|  | Sodium chloride | 530 mM |
|  | Hepes | 530 mM |
|  | BSA | 0.04 % |
| Calcium and Magnesium Free- Artificial Sea Water (CMF-ASW)  (Styfhals et al., 2023) ^42^ | Sodium chloride | 449 mM |
|  | Sodium sulphate • 10H_2_O | 33 mM |
|  | Potassium chloride | 9 mM |
|  | Sodium bicarbonate | 2.15 mM |
|  | TrisHCl | 10 mM |
|  | EGTA | 2.5 mM |
